# Amphiregulin mediates hCG-induced StAR expression and progesterone production in human granulosa cells

**DOI:** 10.1038/srep24917

**Published:** 2016-04-26

**Authors:** Lanlan Fang, Yiping Yu, Ruizhe Zhang, Jingyan He, Ying-Pu Sun

**Affiliations:** 1 Reproductive Medical Center, The First Affiliated Hospital of Zhengzhou University, Zhengzhou, 450052 China

## Abstract

Progesterone plays critical roles in maintaining a successful pregnancy at the early embryonic stage. Human chorionic gonadotropin (hCG) rapidly induces amphiregulin (AREG) expression. However, it remains unknown whether AREG mediates hCG-induced progesterone production. Thus, the objective of this study was to investigate the role of AREG in hCG-induced progesterone production and the underlying molecular mechanism in human granulosa cells; primary cells were used as the experimental model. We demonstrated that the inhibition of EGFR and the knockdown of AREG abolished hCG-induced steroidogenic acute regulatory protein (StAR) expression and progesterone production. Importantly, follicular fluid AREG levels were positively correlated with progesterone levels in the follicular fluid and serum. Treatment with AREG increased StAR expression and progesterone production, and these stimulatory effects were abolished by EGFR inhibition. Moreover, activation of ERK1/2, but not PI3K/Akt, signaling was required for the AREG-induced up-regulation of StAR expression and progesterone production. Our results demonstrate that AREG mediates hCG-induced StAR expression and progesterone production in human granulosa cells, providing novel evidence for the role of AREG in the regulation of steroidogenesis.

After ovulation, luteinizing hormone (LH) stimulates granulosa cells to produce progesterone^1^,^2^. The elevation in progesterone levels that occurs after this LH surge is involved in the regulation of follicular rupture and luteinization, which play critical roles in maintaining a successful pregnancy at the early embryonic stage. Indeed, a deficiency in progesterone secretion during the luteal phase is associated with early miscarriages^3^. Therefore, the precise regulation of progesterone production in granulosa cells is necessary for maintaining normal reproductive functions. Steroidogenesis is a complex process that involves multiple enzymatic reactions^4^. In the ovaries, progesterone is synthesized from cholesterol in the mitochondria of granulosa cells. Once free cholesterol has been transported to the mitochondria, it is transported from the outer to the inner mitochondrial membrane by the steroidogenic acute regulatory protein (StAR), which is well recognized as the key regulatory protein involved in the rate-limiting step of steroidogenesis^5^,^6^. Cholesterol is catalyzed to pregnenolone by the cholesterol side-chain cleavage enzyme complex at the inner mitochondrial membrane. Pregnenolone is then transferred to the cytoplasm and catalyzed to progesterone by 3β-hydroxysteroid dehydrogenase^4^.

Epidermal growth factor receptor (EGFR) and its ligands, which are expressed in female reproductive tissues, have been shown to regulate various important reproductive functions^7^. Epidermal growth factor (EGF), amphiregulin (AREG), betacellulin (BTC), epiregulin (EREG), heparin-binding EGF-like growth factor (HB-EGF) and transforming growth factor-α (TGF-α) all bind to and activate EGFR^8^,^9^. Interestingly, in mouse granulosa cells, the mRNA levels of AREG, BTC and EREG, but not those of EGF, HB-EGF and TGF-α, are rapidly up-regulated by human chorionic gonadotropin (hCG) treatment^10^. Our previous study further confirmed the stimulatory effects of LH/hCG on AREG, BTC and EREG expression in human granulosa cells^11^. Animal studies have demonstrated that inhibition of EGFR activity suppresses LH/hCG-induced ovulation^12^,^13^. In addition, treatment with AREG, BTC and EREG induces cumulus expansion, oocyte maturation and COX-2 expression in mouse follicles^14^. These results suggest that AREG, BTC and EREG play important roles in mediating the biological functions of LH/hCG in granulosa cells.

In granulosa cells, LH/hCG induces StAR expression^1^,^15^. Recent studies by both our group and others have demonstrated that several local growth factors can regulate StAR expression and progesterone production in human granulosa cells in an autocrine/paracrine manner^16^,^17^,^18^,^19^,^20^. After hCG administration, the AREG protein can be detected in the follicular fluid^21^. Although BTC and EREG can also be up-regulated by hCG, the protein levels of BTC and EREG in human follicular fluid are barely detectable^21^. Therefore, AREG is thought to be the most abundant EGFR ligand in human follicular fluid. However, it remains unknown whether AREG mediates LH/hCG-induced StAR expression and progesterone production in human granulosa cells. In addition, it is not yet clear whether AREG regulates StAR expression and progesterone production in human granulosa cells; if it does, the underlying molecular mechanisms remain to be defined.

In the current study, we tested the hypothesis that AREG mediates the hCG-induced up-regulation of StAR expression and progesterone production in human granulosa cells. We found that inhibition of EGFR activity and knockdown of AREG attenuated the hCG-induced up-regulation of StAR expression and progesterone production. Importantly, the protein levels of AREG in the follicular fluid were positively correlated with the progesterone levels in both the serum and follicular fluid. Moreover, using pharmacological inhibitors and specific siRNA, our results further demonstrated that AREG up-regulated StAR expression and progesterone expression via EGFR-mediated activation of the ERK1/2 signaling pathway in human granulosa cells.

## Results

### AREG mediates hCG-induced up-regulation of StAR expression in primary human granulosa cells

**Figure 1:**
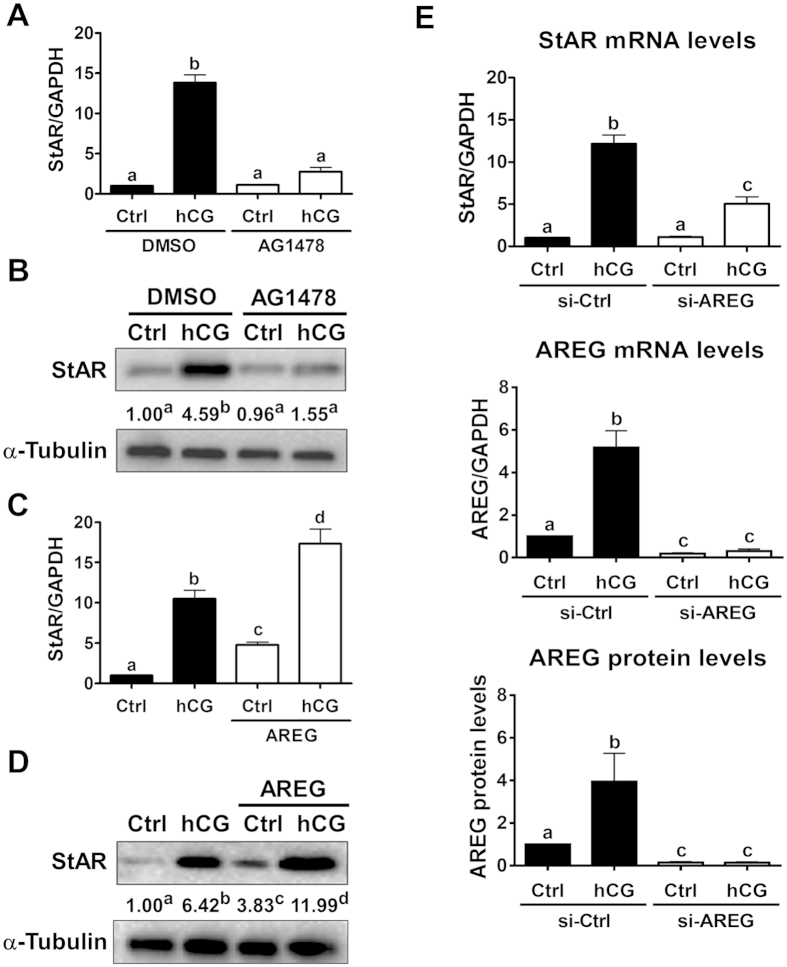
AREG mediates hCG-induced up-regulation of StAR expression in a primary culture of human granulosa cells. (**A,B**) Cells were pretreated with vehicle control (DMSO) or 10 μM of AG1478 for 30 minutes and then treated with 10 IU/mL of hCG for 24 h. The StAR mRNA (**A**) and protein (**B**) levels were examined by RT-qPCR and Western blot analysis, respectively. (**C,D**) Cells were treated with 10 IU/mL of hCG, 100 ng/mL of AREG or 10 IU/mL of hCG in combination with 100 ng/mL of AREG for 24 h. The StAR mRNA (**C**) and protein (**D**) levels were examined by RT-qPCR and Western blot analysis, respectively. (**E**) Cells were transfected with 50 nM of control siRNA (si-Ctrl) or AREG siRNA (si-AREG) for 48 h and then treated with 10 IU/mL of hCG for 24 h. The StAR and AREG mRNA levels were examined by RT-qPCR. The AREG protein levels in the culture media were examined by ELISA. The results are expressed as the mean ± SEM of at least three independent experiments. The numbers under the Western blots represent the summarized quantitative results expressed as the mean of at least three independent experiments. The values without a common letter are significantly different (p < 0.05).

Given the exclusive binding of AREG to EGFR, the function of EGFR was blocked to examine the involvement of AREG in the hCG-induced up-regulation of StAR expression. As shown in Fig. 1A and consistent with previous studies, treatment with hCG for 24 h significantly up-regulated StAR mRNA levels in primary cultures of human granulosa cells. The stimulatory effect of hCG on StAR mRNA levels was abolished by pre-treatment with the specific EGFR tyrosine kinase inhibitor AG1478. Similarly, Western blot results showed that treatment with hCG up-regulated StAR protein levels and that this stimulatory effect was abolished by the inhibition of EGFR function (Fig. 1B). Interestingly, treatment with AREG not only up-regulated the basal mRNA and protein levels of StAR but also enhanced the up-regulation induced by hCG (Fig. 1C,D). To further confirm the involvement of AREG in the hCG-induced up-regulation of StAR expression, an siRNA-mediated knockdown approach was used. As shown in Fig. 1E, the transfection of cells with AREG siRNA significantly down-regulated the basal and hCG-induced mRNA and protein levels of AREG. Importantly, knockdown of AREG attenuated the hCG-induced up-regulation of StAR mRNA levels. These results indicate that AREG is involved in the hCG-induced up-regulation of StAR expression in human granulosa cells.

### AREG mediates hCG-increased progesterone production in primary human granulosa cells

**Figure 2:**
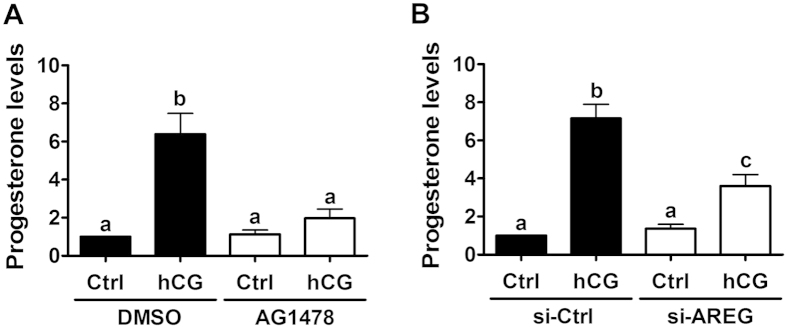
AREG mediates hCG-increased progesterone production in a primary culture of human granulosa cells. (**A**) Cells were pretreated with a vehicle control (DMSO) or 10 μM of AG1478 for 30 minutes and then treated with 10 IU/mL of hCG for 24 h. The progesterone levels in the culture media were examined by ECLIA. (**B**) Cells were transfected with 50 nM of control siRNA (si-Ctrl) or AREG siRNA (si-AREG) for 48 h and then treated with 10 IU/mL of hCG for 24 h. The progesterone levels in the culture media were examined by ECLIA. The results are expressed as the mean ± SEM of at least three independent experiments. The values without a common letter are significantly different (p < 0.05).

StAR is the key regulatory protein controlling the rate-limiting step in progesterone production in granulosa cells. Therefore, we measured the effect of AG1478 on hCG-induced progesterone production. Treatment with hCG increased progesterone production in human primary granulosa cells, and the hCG-induced increase in progesterone production was abolished by pre-treatment with AG1478 (Fig. 2A). In addition, AREG knockdown attenuated hCG-induced progesterone production (Fig. 2B). These results clearly indicate that AREG mediates hCG-induced progesterone production in human granulosa cells.

### Positive correlation between the concentrations of follicular fluid AREG and progesterone

**Figure 3:**
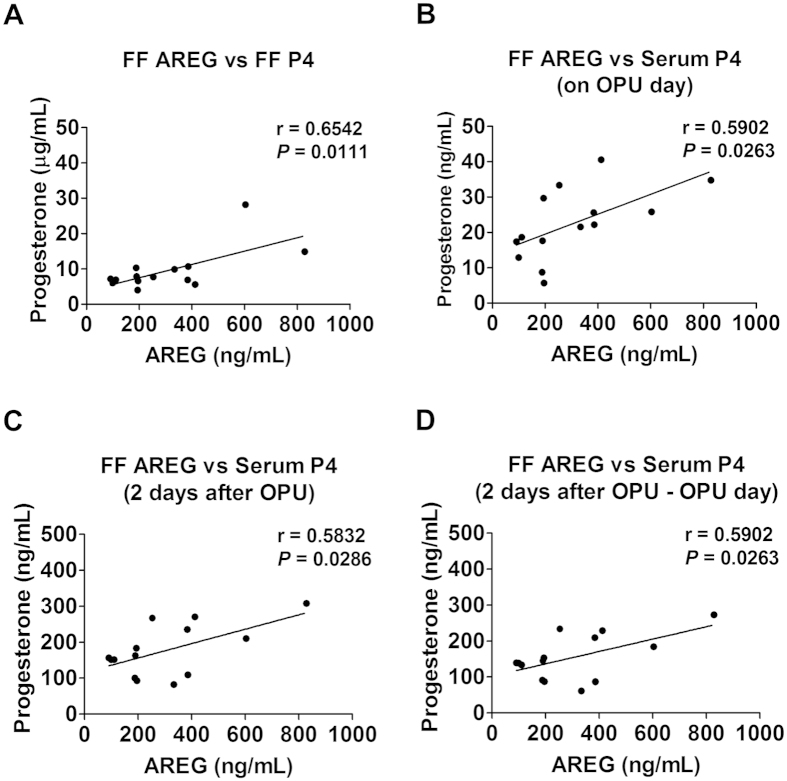
AREG levels in the follicular fluid are positively correlated with progesterone levels in the follicular fluid and serum. The AREG levels in the follicular fluid were examined by ELISA (N = 14). The progesterone levels in the follicular fluid and serum were examined by ECLIA (N = 14). (**A**) The follicular fluid (FF) AREG levels were positively correlated with the levels of progesterone in the FF. (**B**) The FF AREG levels were positively correlated with the levels of progesterone in the serum that were measured on the day of oocyte pick-up (OPU). (**C**) The FF AREG levels were positively correlated with levels of progesterone in the serum that were measured 2 days after OPU. (**D**) The FF AREG levels were positively correlated with the increases in serum progesterone that occurred between the OPU and 2 days after OPU.

To further investigate the relationship between AREG and progesterone, we next measured the concentrations of these two components in follicular fluid samples obtained from 14 IVF patients. In the follicular fluid samples tested, the average concentration of AREG was 304.7 ± 194.5 ng/mL, and the average concentration of progesterone was 9.5 ± 5.6 μg/mL (Fig. 3A). Consistent with the results observed in the cell culture studies, the levels of AREG and progesterone were positively correlated with each other in the follicular fluid (r = 0.6542, *p* = 0.0111). In addition, the levels of AREG in the follicular fluid were positively correlated with the serum levels of progesterone measured on the day of and 2 days after oocyte pick-up (OPU) (Fig. 3B,C). Interestingly, the increase in serum progesterone between the day of OPU and 2 days after OPU were also positively correlated with follicular fluid AREG levels (Fig. 3D). These clinical results clearly demonstrate the stimulatory effect of AREG on progesterone production in human granulosa cells.

### AREG up-regulates StAR expression in primary human granulosa cells

**Figure 4:**
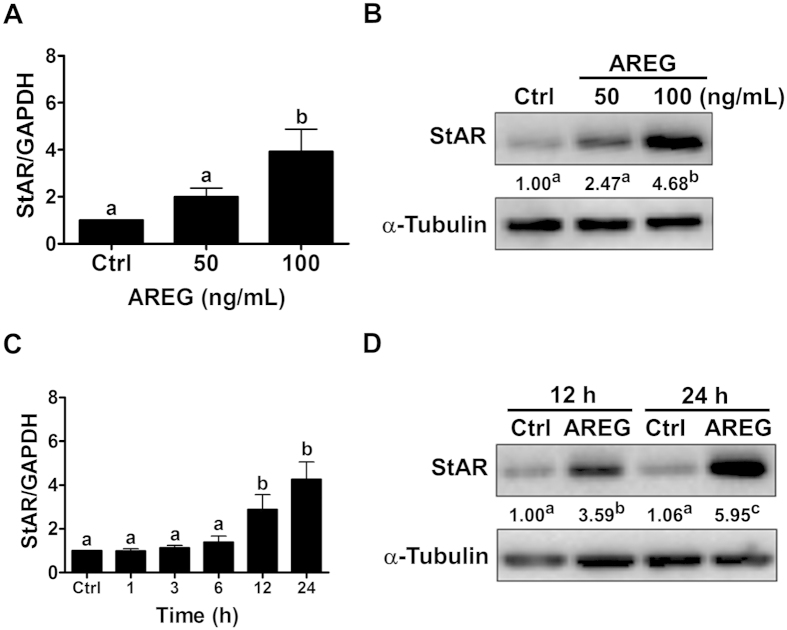
AREG up-regulates StAR expression in primary human granulosa cells. (**A,B**) Cells were treated with 50 or 100 ng/mL of AREG for 24 h, and the mRNA (**A**) and protein (**B**) levels of StAR were examined by RT-qPCR and Western blot analysis, respectively. (**C,D**) Cell were treated with 100 ng/mL of AREG for different periods of time, and the mRNA (**C**) and protein (**D**) levels of StAR were examined by RT-qPCR and Western blot analysis, respectively. The level of StAR mRNA at each time point was normalized to the GAPDH mRNA level at the same time point. The results are expressed as the mean ± SEM of at least three independent experiments. The numbers under the Western blots represent the summarized quantitative results expressed as the mean of at least three independent experiments. The values without a common letter are significantly different (p < 0.05).

Notably, AREG not only enhanced the hCG-induced up-regulation of StAR expression but also up-regulated basal StAR expression in human granulosa cells. Given the strong stimulatory effect of AREG on StAR expression, we next examined its underlying molecular mechanisms, which have not been previously reported. Treating primary human granulosa cells with 50 ng/mL of AREG for 24 h slightly increased StAR mRNA levels, but no significant difference was observed. However, treating cells with 100 ng/mL of AREG significantly up-regulated StAR mRNA levels (Fig. 4A). Western blot results confirmed the stimulatory effect of AREG on StAR protein levels (Fig. 4B). We and other groups have shown that the concentration of AREG in human follicular fluid can exceed 100 ng/mL^10^,^22^. Therefore, 100 ng/mL of AREG was used in subsequent experiments. AREG treatment up-regulated StAR mRNA levels in a time-dependent manner (Fig. 4C). Similar to the RT-qPCR results, StAR protein levels were up-regulated after 12 and 24 h of AREG treatment (Fig. 4D).

### EGFR is required for the AREG-induced up-regulation of StAR expression

**Figure 5:**
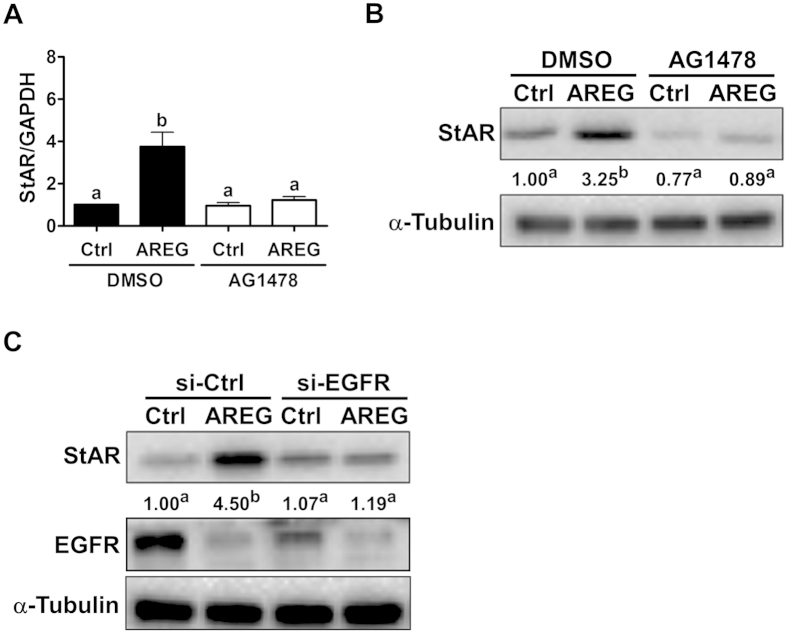
EGFR is required for AREG-induced StAR expression in primary human granulosa cells. (**A,B**) Cells were pretreated with a vehicle control (DMSO) or 10 μM AG1478 for 30 minutes and then treated with 100 ng/mL of AREG for 24 h. The StAR mRNA (**A**) and protein (**B**) levels were examined by RT-qPCR and Western blot analysis, respectively. (**C**) Cells were transfected with 50 nM of control siRNA (si-Ctrl) or EGFR siRNA (si-EGFR) for 48 h and treated with 100 ng/mL of AREG for 24 h. The StAR protein levels were examined by Western blot analysis. The results are expressed as the mean ± SEM of at least three independent experiments. The numbers under the Western blots represent the summarized quantitative results expressed as the mean of at least three independent experiments. The values without a common letter are significantly different (p < 0.05).

To investigate whether EGFR is required for the AREG-induced up-regulation of StAR expression, AG1478 was used to block the function of EGFR. Treatment with AREG up-regulated StAR mRNA levels, and this stimulatory effect was abolished by pre-treatment with AG1478 (Fig. 5A). Western blot analyses showed similar results (Fig. 5B). To avoid any off-target effects of the pharmacological inhibitor and to further confirm the requirement of EGFR in the AREG-induced up-regulation of StAR expression, EGFR siRNA was used to knockdown endogenous EGFR expression. As shown in Fig. 5C, transfection with EGFR siRNAs significantly down-regulated EGFR protein levels. Moreover, knockdown of EGFR abolished the AREG-induced up-regulation of StAR protein levels. The binding of EGFR ligands to EGFR rapidly induces the clustering and internalization of the ligand-receptor complexes, ultimately resulting in lysosomal degradation of both EGF and its receptor^23^. As shown in Fig. 5C, our data support this process and show that EGFR was down-regulated in primary human granulosa cells in response to AREG treatment.

### The ERK1/2 signaling pathway is involved in the AREG-induced up-regulation of StAR expression

**Figure 6:**
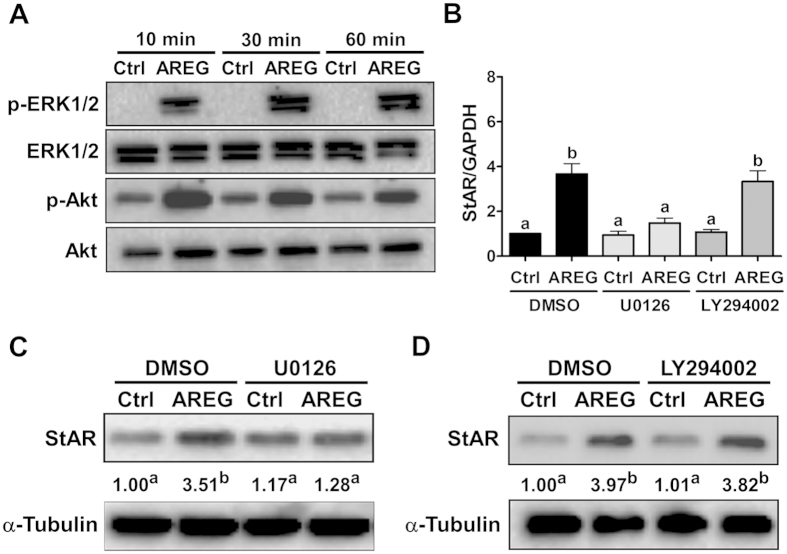
ERK1/2 signaling mediates AREG-induced StAR expression in primary human granulosa cells. (**A**) Cells were treated with 100 ng/mL of AREG for 10, 30 or 60 min. Phosphorylation of ERK1/2 and Akt were determined by Western blot analysis using antibodies that are specific for the phosphorylated forms of ERK1/2 (p-ERK1/2) and Akt (p-Akt). Membranes were stripped and re-probed with antibodies detecting total ERK1/2 and Akt. (**B**) Cells were pretreated with a vehicle control (DMSO), 10 μM of U0126 or 10 μM of LY294002 for 30 minutes and then treated with 100 ng/mL of AREG for 24 h. The StAR mRNA levels were examined by RT-qPCR. (**C,D**) Cells were pretreated with a vehicle control (DMSO), 10 μM of U0126 (**C**) or 10 μM of LY294002 (**D**) for 30 minutes and then treated with 100 ng/mL of AREG for 24 h. The StAR protein levels were examined by Western blot analysis. The results are expressed as the mean ± SEM of at least three independent experiments. The numbers under the Western blots represent the summarized quantitative results expressed as the mean of at least three independent experiments. The values without a common letter are significantly different (p < 0.05).

We have previously shown that AREG activates the ERK1/2 and PI3K/Akt signaling pathways in an immortalized human granulosa cell line, SVOG^11^. However, whether the same regulatory mechanism is present in primary human granulosa cells remains to be determined. As shown in Fig. 6A, similar to previous results obtained from SVOG cells, treatment with AREG activated both the ERK1/2 and Akt signaling pathways in primary human granulosa cells. Next, we used specific inhibitors of MEK and PI3K to determine which pathways are required for AREG-induced StAR up-regulation. As shown in Fig. 6B, pre-treatment with the MEK inhibitor U0126 abolished the AREG-induced up-regulation of StAR mRNA, whereas pretreatment with the PI3K inhibitor LY294002 had no effect. Western blot results further confirmed that ERK1/2, but not PI3K/Akt, signaling was required for the AREG-induced up-regulation of StAR protein levels in primary human granulosa cells (Fig. 6C,D).

### The ERK1/2 signaling pathway is involved in AREG-induced progesterone production

**Figure 7:**
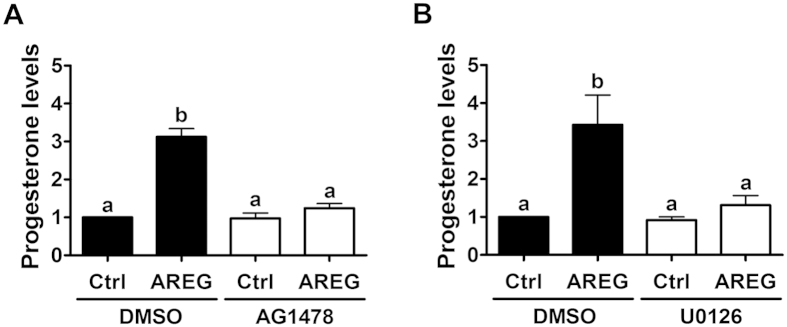
ERK1/2 signaling mediates AREG-induced progesterone production in primary human granulosa cells. (**A**) Cells were pretreated with a vehicle control (DMSO) or 10 μM of AG1478 for 30 minutes and then treated with 100 ng/mL of AREG for 24 h. The progesterone levels in the culture media were examined by ECLIA. (**B**) Cells were pretreated with a vehicle control (DMSO) or 10 μM of U0126 for 30 minutes and then treated with 100 ng/mL of AREG for 24 h. The progesterone levels in the culture media were examined by ECLIA. The results are expressed as the mean ± SEM of at least three independent experiments. The values without a common letter are significantly different (p < 0.05).

We next examined the influence of AREG on progesterone production in primary human granulosa cells. Cells were treated with AREG for 24 h, and the culture media were collected; the levels of progesterone in the culture media were examined by ECLIA. As shown in Fig. 7A, treatment with AREG significantly increased progesterone production, and this increase was completely abolished by the inhibition of EGFR. In addition, the inhibition of ERK1/2 signaling by U0126 also abolished the stimulatory effect of AREG on progesterone production (Fig. 7B). These results indicate that ERK1/2 signaling is required for AREG-induced increases in progesterone production in primary human granulosa cells.

## Discussion

Ovarian granulosa cells are essential for normal oocyte development and steroid hormone production. Therefore, a better understanding of the regulation of progesterone production in granulosa cells will greatly facilitate future clinical translational endeavors. In the present study, we demonstrate for the first time that AREG mediates hCG-induced StAR expression and progesterone expression in human granulosa cells. In addition, our clinical data show that follicular fluid AREG levels are positively correlated with serum and follicular fluid progesterone levels, which further supports our findings that AREG stimulates progesterone production in the ovaries. Moreover, our results demonstrate that the EGFR-mediated activation of ERK1/2 signaling is required for AREG-induced StAR expression and progesterone production in human granulosa cells.

The elevation in progesterone levels that occurs after the LH surge is involved in the regulation of follicular rupture and luteinization, which play critical roles in maintaining a successful pregnancy at the early embryonic stage, and deficiency in progesterone secretion during the luteal phase is associated with early miscarriages^3^. Therefore, the production of progesterone must be tightly regulated to ensure a successful pregnancy. In the present study, we show that AREG mediated hCG-induced StAR expression and progesterone production in human granulosa cells. In addition, the increase in serum progesterone between the day of OPU and 2 days after OPU was positively correlated with the levels of AREG in the follicular fluid. These results suggest that the expression of AREG in the follicular fluid may be used as an indicator to predict the levels of progesterone, which are important for a successful pregnancy. Interestingly, a recent study shows that AREG mRNA levels in mural and cumulus granulosa cells are significantly higher in pregnant women than in non-pregnant women. Importantly, LH/hCG-induced AREG expression is positively correlated with the number of oocytes retrieved and with good-quality embryos^21^,^24^. Therefore, the expression levels of AREG may also be used as a marker for oocyte developmental competency.

We have previously shown that LH/hCG up-regulates AREG, BTC and EREG expression in human granulosa cells^11^. However, the levels of BTC and EREG are very low and are barely detectable in human follicular fluid^21^. Our results show that inhibition of EGFR abolished hCG-induced StAR expression, which indicates that EGFR ligands mediate the stimulatory effect of hCG on StAR expression. To directly examine the involvement of AREG in the hCG-induced up-regulation of StAR expression, we used an siRNA-mediated knockdown approach to block the expression of both endogenous and hCG-induced AREG expression. Unexpectedly, AREG knockdown only partially attenuated hCG-induced StAR up-regulation. These results suggest that other EGFR ligands, such as BTC and EREG, are also involved in the StAR up-regulation induced by hCG treatment. AREG is the most abundant EGFR ligand in human follicular fluid^21^. However, our previous study demonstrates that BTC and EREG are more potent than AREG in stimulating COX-2 expression and PGE2 production in human granulosa cells^11^. Therefore, BTC and EREG could also play important roles in mediating the hCG-induced up-regulation of StAR expression. Additional studies will be needed to address this hypothesis.

The function of granulosa cells is well known to be regulated by endocrine regulators such as FSH and LH. To date, many studies have demonstrated that members of the transforming growth factor-β (TGF-β) superfamily, including TGF-β1, activins, bone morphogenetic proteins (BMPs) and growth and differentiation factors (GDFs), are expressed in the ovaries and are able to regulate StAR expression and progesterone production in human granulosa cells in an autocrine and/or paracrine fashion^16^,^17^,^18^,^19^,^20^,^25^,^26^,^27^,^28^,^29^. These results indicate that the local production of hormonal factors also plays an important role in regulating the functions of granulosa cells. Our group and others have reported that LH/hCG stimulation up-regulates AREG expression in human granulosa cells^11^,^24^,^30^,^31^,^32^. Moreover, AREG protects human granulosa cells from serum starvation-induced apoptosis, and acts as a pro-survival LH mediator in the human corpus luteum^32^. Taken together, these results indicate that AREG is an important regulator of ovarian granulosa cells after ovulation.

Although treatment with AREG has been shown to increase progesterone production in human granulosa cells obtained from immature follicles during IVF procedures^30^,^32^, the molecular mechanisms mediating this action of AREG remain unknown. Depending on the stage of the menstrual cycle during which the granulosa cells were collected, the primary culture of human granulosa cells used in the present study provide an ideal *in vitro* model for studying the hormonal effects on ovulation- and corpus luteum-related functions. Our results using this cell model show that EGFR-mediated activation of ERK1/2 signaling was required for the AREG-induced up-regulation of StAR expression and progesterone production. AREG has been shown to bind exclusively to EGFR^9^. Upon AREG binding, many intracellular signaling pathways, including MAPK, PI3K/Akt, STAT and mTOR, are activated and mediate the AREG/EGFR-regulated cellular functions^33^. To date, various signaling pathways have been reported to be involved in the regulation of StAR expression, among which ERK1/2 signaling is a well-characterized pathway that mediates StAR expression^34^. Interestingly, in human granulosa cells, the activation of ERK1/2 is involved in gonadotropin-stimulated StAR expression^35^,^36^. In contrast, ERK1/2 activation is required for TGF-β1- and GDF8-inhibited StAR expression^17^,^18^. These results suggest that the role of ERK1/2 signaling in the regulation of StAR expression is context dependent and that in addition to ERK1/2 signaling, other signaling pathways or co-factors are also involved in regulating StAR expression.

In summary, the present study demonstrates that AREG mediates the hCG-induced up-regulation of StAR expression and progesterone production in human granulosa cells. In addition, our results show that the levels of AREG in the follicular fluid are positively correlated with progesterone levels in the serum and follicular fluid. Moreover, we show that the EGFR-mediated activation of ERK1/2 is required for the AREG-induced up-regulation of StAR expression and progesterone production. These results reveal the physiological roles and molecular mechanisms of AREG in the regulation of StAR expression and progesterone production in human granulosa cells, which might help in developing new strategies for the treatment of clinical infertility.

## Materials and Methods

### Study Subjects

Human serum and follicular fluid samples were obtained from 14 infertile women treated with *in vitro* fertilization (IVF) or an intracytoplasmic sperm injection (ICSI). According to our preliminary data, the correlation coefficient for AREG and progesterone in the follicular fluid was estimated to be 0.7, which indicates that to detect a difference in the correlation coefficient with an α = 0.05 and β = 90%, 14 patients would needed for this study. Informed patient consent was obtained from all participants. The study received approval and was carried out in accordance with the approved guidelines from the Zhengzhou University Research Ethics Board. The experimental protocols were approved by the Zhengzhou University Research Ethics Board. All 14 patients were between the ages of 20 and 35 and had normal menstrual cycles. Their causes of infertility were tubal obstruction or male infertility. Patients with polycystic ovarian syndrome, endometriosis, diminished ovarian reserves, chromosomal abnormalities or hydrosalpinx were excluded from this study. The clinical characteristics of the 14 patients were summarized in Table 1.

### Controlled ovarian hyperstimulation protocols

All patients were treated with a standard long protocol^37^. At the mid-luteal phase, the gonadotropin-releasing hormone (GnRH) agonist triptorelin (0.1 mg) (Ipsen Pharma Biotech, France) was administered subcutaneously (s.c.) daily. Approximately 14 days after the first injection of the GnRH agonist, recombinant FSH (Gonal-F; Merck, Germany) at a dosage of 150–300 IU was administered daily. Human menopausal gonadotropin (hMG; Livzon, China) was also used if needed. hCG (Livzon) was injected when at least three follicles had reached 18 mm. Oocyte retrieval was scheduled approximately 34–36 h after hCG injection via transvaginal ultrasound-guided follicular aspiration.

### Collection of blood and follicular fluid

Blood samples were obtained by venipuncture on the day of oocyte pick-up (OPU day) and 2 days after OPU. After collection, the serum was stored at −80 °C until it was assayed. The follicular fluid was collected when the oocytes were retrieved. Only the first follicular fluid aspirate without blood or flushing solution was used for analysis. After 10 min of centrifugation at 1200 rpm, the supernatant was stored at −80 °C until it was assayed.

### Primary culture of human granulosa cells

The primary human granulosa cells were purified using density centrifugation from follicular aspirates collected from women undergoing oocyte retrieval as previously described^38^,^39^. The follicular fluid was collected in a sterile container when the oocytes were retrieved. Cells were cultured in a humidified atmosphere containing 5% CO_2_ and 95% air at 37 °C in Dulbecco’s Modified Eagle Medium/nutrient mixture F-12 Ham medium (DMEM/F-12; Gibco, Grand Island, NY) supplemented with 10% charcoal/dextran-treated FBS (HyClone, Logan, UT), 100 U/mL of penicillin and 100 μg/mL of streptomycin sulfate (Boster, China). For all of the treatment experiments (hCG and AREG), the cells were placed in 12-well plates at densities of 5 × 10^4^ cells/cm^2^ with 1 mL of culture medium. After 5 days of cultivation, the medium was changed to a medium containing 0.5% charcoal/dextran-treated FBS and the cells were cultured for another 24 h. All treatments were performed in medium containing 0.5% charcoal/dextran-treated FBS.

### Antibodies and reagents

Monoclonal mouse anti-phospho-ERK1/2 (Thr185/Tyr187) antibody was obtained from Proteintech (Chicago, IL). Polyclonal rabbit anti-phospho-Akt (Ser473), anti-ERK1/2, and anti-Akt antibodies were obtained from Cell Signaling Technology (Danvers, MA). Polyclonal rabbit anti-StAR and anti-EGFR antibodies were obtained from Santa Cruz Biotechnology (Santa Cruz, CA). Monoclonal mouse anti-α-tubulin antibody was obtained from CMCTAG (Milwaukee, WI). Horseradish peroxidase-conjugated goat anti-rabbit and goat anti-mouse IgGs were obtained from Abcam (Cambridge, MA). Recombinant human AREG was obtained from R&D Systems (Minneapolis, MN). hCG was obtained from Livzon. AG1478, U0126 and LY294002 were obtained from Sigma-Aldrich Corp (Shanghai, China).

### Reverse transcription quantitative real-time PCR (RT-qPCR)

Total RNA was extracted with the RNeasy Plus Mini Kit (QIAGEN, Shanghai, China) according to the manufacturer’s instructions. RNA (2 μg) was reverse-transcribed into first-strand cDNA with the GoldScript one-step RT-PCR Kit (Applied Biosystems Grand Island, NY). Each 20 μL qPCR reaction volume contained 1X SYBR Green PCR Master Mix (Applied Biosystems), 20 ng of cDNA and 250 nM of each specific primer. The primers used were 5′-AAA CTT ACG TGG CTA CTC AGC ATC-3′ (sense) and 5′-GAC CTG GTT GAT GCT CTT G-3′ (antisense) for StAR and 5′-ATG GAA ATC CCA TCA CCA TCT T-3′ (sense) and 5′-CGC CCC ACT TGA TTT TGG-3′ (antisense) for GAPDH. The qPCR was performed on an Applied Biosystems 7500 Real-Time PCR System equipped with 96-well optical reaction plates. The specificity of each assay was validated using a dissociation curve analysis and agarose gel electrophoresis of the PCR products. Assay performance was validated by evaluating the amplification efficiencies with calibration curves and ensuring that the plot of the log input amount *vs*. Ct had a slope <|0.1|. Alternatively, TaqMan gene expression assays for AREG and GAPDH (Applied Biosystems) were performed in triplicate on corresponding cDNA samples. The PCR parameters for the reaction were 50 °C for 2 min, 95 °C for 10 min, and 40 cycles of 95 °C for 15 sec and 60 °C for 1 min. Three separate experiments were performed on different cultures, and each sample was assayed in triplicate. A mean value was used for the determination of mRNA levels using the comparative Ct (2^−∆∆Ct^) method with GAPDH as the reference gene.

### Western blot

Cells were lysed in cell lysis buffer (Cell Signaling Technology). Equal amounts of protein were separated by SDS polyacrylamide gel electrophoresis and transferred onto PVDF membranes. After 1 h of blocking with 5% non-fat dry milk in Tris-buffered saline (TBS), the membranes were incubated overnight at 4 °C with primary antibodies, which were diluted in 5% non-fat milk/TBS. Following primary antibody incubation, the membranes were incubated with the appropriate HRP-conjugated secondary antibody. Immunoreactive bands were detected using an enhanced chemiluminescent substrate (Bio-Rad Laboratories; Shanghai, China). The chemiluminescent blots were imaged with the ChemiDoc MP Imager (Bio-Rad Laboratories).

### AREG and progesterone measures

AREG levels in the follicular fluid were measured using an enzyme-linked immunosorbent assay (ELISA). The human AREG ELISA Kit (R&D Systems) was used in accordance with the manufacturer’s protocol. Progesterone levels in the serum, culture media and human follicular fluid samples were measured using an electrochemiluminescence immunoassay (ECLIA). Progesterone ECLIA Kit (Roche Diagnostics, Germany) was used in accordance with the manufacturer’s protocol. AREG and progesterone levels in the culture media were normalized to the protein concentrations of the cell lysates. Normalized progesterone values in the culture media collected from the treatments were presented as relative values based on a comparison with the control treatment.

### Small interfering RNA (siRNA) transfection

To knock down endogenous AREG or EGFR expression, cells were transfected with 50 nM Silencer Select Validated siRNAs targeting the specific gene (Thermo Fisher Scientific, Grand Island, NY) using the Lipofectamine RNAiMAX transfection reagent (Invitrogen, Grand Island, NY). The Silencer Select Negative Control (Thermo Fisher Scientific) was used as the transfection control. The knockdown efficiency was determined using RT-qPCR or Western blot analysis.

### Statistical analysis

The results are presented as the mean ± SEM of at least three independent experiments. Multiple comparisons were analyzed using a one-way ANOVA followed by Tukey’s multiple comparison test. A significant difference was defined as *p* < 0.05.

## Additional Information

**How to cite this article**: Fang, L. *et al*. Amphiregulin mediates hCG-induced StAR expression and progesterone production in human granulosa cells. *Sci. Rep*. **6**, 24917; doi: 10.1038/srep24917 (2016).

## Figures and Tables

**Table 1:** The general characteristics of the infertile patients.

Characteristics (N = 14)	Results
AGE	29.1 ± 2.9
BMI	22.5 ± 3.4
Basal FSH (mIU/mL)	6.0 ± 0.7
Basal LH (mIU/mL)	7.0 ± 1.6
Basal E2 (pg/mL)	68.1 ± 59.07
Basal P4 (ng/mL)	0.9 ± 0.5
Basal T (ng/mL)	0.4 ± 0.2
Basal PRL (ng/mL)	19.8 ± 8.0
The number of antral follicle	17.3 ± 5.4
The days of r-FSH administered	10.5 ± 1.2
The total dose of r-FSH administered (IU)	1425.7 ± 347.3
The number of oocytes retrieved	15.6 ± 6.8
P4 in the FF (μg/mL)	9.5 ± 5.6
AREG in the FF (ng/mL)	304.7 ± 194.5

Abbreviation: BMI, body mass index; FSH, follicle stimulating hormone; LH, luteinizing hormone; E2, estradiol; P4, progesterone; T, testosterone; PRL, prolactin; r-FSH, recombinant FSH; AREG, amphiregulin; FF, follicular fluid. Data are presented as mean ± SEM.
